# Genetic diversity, population structure and quality-trait associations in *Blumea balsamifera* revealed by EST-SSR markers

**DOI:** 10.1371/journal.pone.0328403

**Published:** 2025-07-17

**Authors:** Mei Huang, Xin Li, Zhenxia Chen, Dan Wang, Kai Wang, Xuan Hu, Xiaolu Chen, Lingliang Guan, Hongrui Zhang, Yuxin Pang, Fulai Yu

**Affiliations:** 1 Tropical Crops Genetic Resources Institute, Chinese Academy of Tropical Agricultural Sciences, Haikou, China; 2 Key Laboratory of Biology and Cultivation of Herb Medicine (Haikou), Ministry of Agriculture and Rural Affairs, Haikou, China; 3 Hainan Provincial Engineering Research Center for Blumea balsamifera, Haikou, China; 4 College of Agriculture, Henan Agricultural University, Zhengzhou, China; 5 School of Pharmacy, Guizhou University of Traditional Chinese Medicine, Guiyang, China; 6 State Key Laboratory of Tropical Crop Breeding, Sanya, China; 7 Key Laboratory of Tropical Crops Germplasm Resources Genetic Improvement and Innovation of Hainan Province, Haikou, China; National Cheng Kung University, TAIWAN

## Abstract

*Blumea balsamifera* is a widely used ancient medicinal herb in tropical and subtropical Asia. Studying the genetic diversity of *B. balsamifera* germplasm resources and developing molecular markers related to the content of active constituents in *B. balsamifera* are of great significance for breeding varieties with high active ingredient content. In this study, the contents of 6 key active constituents were determined in 51 *B. balsamifera* accessions. EST-SSR markers were used to study their genetic diversity. At the same time, genetic structure analysis and linkage disequilibrium analysis were performed. On this basis, association analysis between EST-SSR markers and active ingredient content was performed. Quantitative analysis revealed substantial variation in six bioactive compounds, with coefficients of variation (*CV*) exceeding 50% across all metabolites. This result revealed its suitability for association analysis. Genetic diversity analysis revealed a total of 102 alleles amplified from 22 pairs of primers, with effective alleles accounting for 53.52%. The average polymorphism information content was 0.488, with 9 pairs of primers exhibiting high polymorphism (*PIC* > 0.5), 11 pairs of primers showing moderate polymorphism (0.25 < *PIC* < 0.5). The proposed primers demonstrated strong effectiveness and good polymorphism. The average *Nei* diversity index and Shannon information index were 0.542 and 1.023, respectively, indicating a high level of genetic diversity within the population. UPGMA clustering and population structure analyses classified the 51 accessions into four distinct groups correlated with geographical origins. LD analysis indicated over 50% of marker pairs showed substantial linkage (D’ > 0.5), validating the suitability of this germplasm set for association mapping. Association analysis between quality traits and EST-SSR markers showed that, in both GLM and MLM models, Bbf137 and Bbf377 were found to be associated with 3,3’,5,7-tetrahydroxy-4’- methoxyflavanone (TMF, *P* < 0.05) and Blumeatin (*P* < 0.01) respectively. These findings lay a robust foundation for future breeding strategies and genetic enhancement of *B. balsamifera.*

## Introduction

*Blumea balsamifera* (L.) DC, a member of the Blumea genus in the Asteraceae family, is widely distributed in China, Malaysia, Philippines, Thailand, and Vietnam. It has been traditionally used in tropical and subtropical regions of Asia for treating various conditions, including eczema, dermatitis, rheumatism, and skin lesions [1]. Notably, it is the sole plant source of Aipian, a compound recorded in the Pharmacopoeia of the People’s Republic of China (2020 version). In recent years, the medicinal value of *B. balsamifera* has attracted significant attention, leading to extensive studies on its chemical composition, particularly focusing on volatile oils and flavonoids [2]. *l*-borneol is one of the major components of *B. balsamifera* essential oil, constituting over 85% of Aipian. *l*‑Borneol is the pharmacopeial quality‑control marker for Aipian. It exhibits pharmacological activities such as tissue repair, antibacterial, and anti-inflammatory effects. Additionally, key flavonoids found in *B. balsamifera*, including 3,3’,5,7-tetrahydroxy-4’-methoxyflavanone (TMF), Eriodictyol, 3,3’,5-Trihydroxy-4’,7- dimethoxyflavanone (TDF), Blumeatin, and Sakuranetin, possess antibacterial, antitumor, and antioxidant properties. These compounds are frequently used as quality control markers in the quality evaluation of *B. balsamifera* [1,3,4]. As the exclusive plant source of Aipian, there is a growing interest among breeders to improve its quality. Germplasm resources serve as the foundation and essential materials for breeding, making the study of genetic diversity crucial for efficient utilization and innovation of germplasm resources [5]. In the early stages, the research team conducted a comprehensive analysis of *B. balsamifera* germplasm resources and observed a significant decline in recent years. This decline can be attributed to the impact of industrial development, unregulated harvesting practices, and other contributing factors. Consequently, the reduction in germplasm resources has resulted in inconsistent quality and challenges in controlling the output and quality of medicinal materials [6]. In addition, the limited genomic information available for *B. balsamifera* hampers the effective utilization of its germplasm resources [7,8]. Therefore, studying the genetic diversity of germplasm resources is crucial for their efficient utilization and innovative breeding.

DNA molecular markers serve as important tools for studying genetic diversity in germplasm resources and molecular-assisted breeding. They primarily include random amplified polymorphic DNA (RAPD) [9], Sequence-Related Amplified Polymorphism (SRAP) [10], simple sequence repeats (SSR), Inter-Simple Sequence Repeat(ISSR) [11], and so on. With the advancements in molecular marker technology, these markers have been widely employed for analyzing plant genetic diversity, fingerprints and quantitative trait loci (QTL) mapping [12–14]. Among them, simple sequence repeats (SSRs) are codominant markers known for their stability, repeatability, and polymorphism, making them one of the most extensively used markers in molecular studies. Previous studies have utilized RAPD, SRAP, and AFLP markers to investigate the clonal diversity and genetic diversity of *B. balsamifera* populations [7,8]. However, no reports have explored the use of SSR markers for association analysis of active components in the germplasm resources of *B. balsamifera*. Previous studies have shed light on the clonal diversity of *B. balsamifera* populations using various molecular markers. Pang [8] employed RAPD technology to investigate clonal diversity in four wild populations, revealing a high level of clonal diversity in *B. balsamifera*. Similarly, Zhang [7] utilized widely-used SRAP and AFLP markers to compare and analyze the genetic diversity of 35 *B. balsamifera* resources. Their findings demonstrated the advantages of AFLP markers, including a high molecular marker index, making them suitable for evaluating the genetic diversity of *B. balsamifera*.

While association analysis, also known as association mapping, has been extensively applied in crops such as corn, bean, and Tobacco [14–16], its application in the evaluation of active components in Chinese herbal medicine germplasm resources, particularly *B. balsamifera*, remains unexplored. Association analysis is a method that utilizes natural populations or germplasm resources to detect significant associations between markers in linkage disequilibrium and target traits [17–19]. Thus, the application of EST-SSR markers for association analysis of active components in *B. balsamifera* germplasm resources presents an unexplored research area.

Here, we analyzed six active ingredients in 51 *B. balsamifera* accessions collected from major distribution regions across China. Using EST-SSR markers, the genetic diversity and relationships of *B. balsamifera* were analyzed, and molecular markers associated with active ingredient contents were identified.These findings can provide a theoretical basis for molecular marker-assisted breeding of specialized varieties with high content of effective ingredients.

## Materials and methods

### Plant material

The 51 *B. balsamifera* accessions were collected from Guizhou (18), Guangxi (11), Guangdong (2), and Hainan (20) in China (Table 1). The germplasm was classified into wild and cultivated types based on field habitat and the presence/absence of visible human management traces (irrigation, pruning patterns). All accessions were cultivated at the germplasm resource nursery of *B. balsamifera* in Danzhou, China. The germplasm characteristics were stable and well-documented.

**Table 1:** Source of 51 *B. balsamifera* accessions.

Origin	Number	Germplasm type
**Luodian, Guizhou**	1–6, 11	Wild
**Luodian, Guizhou**	7, 10, 12, 26 - 31	Cultivated
**Wangmo, Guizhou**	49	Wild
**Zhenfeng, Guizhou**	45	Wild
**Longlin, Guangxi**	35	Wild
**Tianlin, Guangxi**	36–40, 43	Wild
**Xilin, Guangxi**	41, 42, 44	Wild
**Nanning, Guangxi**	46	Cultivated
**Guangzhou, Guangdong**	47, 48	Cultivated
**Danzhou, Hainan**	8, 9, 13-19, 25	Cultivated
**Qiongzhong, Hainan**	20–24, 34	Wild
**Tunchang, Hainan**	32, 33	Wild
**Wanning, Hainan**	51	Wild
**Wuzhishan, Hainan**	50	Wild

### Genotyping

Fresh young leaves from samples were used for DNA extraction by using the magnetic bead method genomic DNA extraction kit (Tiangen Biotech) with automated workstation. The quality of DNA was detected by 1% agarose gel electrophoresis, and the concentration and purity of nucleic acid were measured using a NanoDrop 8000 ultra-micro spectrophotometer, with an absorbance ratio of A260/A280 = 1.8–2.0. The development of EST-SSR markers was based on transcriptome data from our laboratory. MISA software was used to analyze the transcriptome sequencing data and identify potential SSR loci. Primer 6.0 software was used for primer design, and the SSR primers were verified against the Unigene library through BLAST analysis. Initial synthesis of 384 primer pairs was guided by prior sequencing data from our research group. Primary screening employing pooled DNA from 10 genetically diverse *B. balsamifera* accessions identified 87 primer pairs meeting stringent selection criteria: clean capillary electrophoresis peak morphology, ≥ 3 alleles per locus, and PIC values >0.3. Subsequent validation through individual-accession PCR confirmed 22 core primer pairs demonstrating electrophoretic band clarity, high polymorphism, and robust amplification stability (Table 2).

**Table 2:** Information of 22 EST-SSR primers.

Primers	Repeat motifs	Primer sequence (5’ – 3’)	Size of bands Amplified (bp)	Fluorescent labelled
**Bbf001**	(CT)_7_	F:TGTCACTGCACCACCTTTG	342 - 346	HEX
		R:GGCACAAACGGCTATGGTC		
**Bbf020**	(AAG)_4_	F:CCCTCGCCCTATTTCACCG	222 - 226	FAM
		R:CATCCCGGCGATCCTTCC		
**Bbf041**	(CT)_7_	F:CTGTCAGCACCATTTCCCG	285 - 289	HEX
		R:GTGGAACAACGCTATCTGGC		
**Bbf065**	(CGG)_5_	F:CGCCGTAGCTGAAATCGTG	263 - 269	FAM
		R:CGCTTGCGAACCCATAGTC		
**Bbf068**	(AG)_8_	F:TGTTGCATTCCATGATCCACC	311 - 319	FAM
		R:CTTTACTGAAGCTGTGCAACTC		
**Bbf070**	(GT)_6_	F:CTGGTCTTGTAAGCGGTGC	321 - 333	FAM
		R:AGCACATGGTTTCCTGGATTAG		
**Bbf103**	(ATT)_4_	F:ACGGCACATGGACCAAATC	255 - 266	FAM
		R:TCCATCCAATTCGTTTCCTCG		
**Bbf106**	(AC)_8_	F:GGGCAAATCCACTTTGGGAG	222 - 240	HEX
		R:TCATGTCGGAAATAGAAGCGG		
**Bbf137**	(AAAC)_4_	F:GAGTGTTGCTCATCCAGGC	176 - 188	HEX
		R:TGCAAGTCATCAGGCAAAGG		
**Bbf140**	(ACG)_4_	F:AGCATTCTGGCAGCTCAAC	297 - 306	HEX
		R:CAGCAGGCATTGAAGACGG		
**Bbf146**	(CT)_10_	F:GCAAAGGAGTGGTGAAGCC	248 - 256	ROX
		R:TGAACACGGATTTGGGCAAG		
**Bbf156**	(AAG)_4_	F:TCCACTAGGCACGGCATAC	222 - 228	HEX
		R:TCGTTTCTTGAGCGTCACC		
**Bbf161**	(CT)_8_	F:TCATCAAAGCTGCAACCCG	275 - 281	FAM
		R:GTTCGGTTTCGCCTAGTGG		
**Bbf193**	(ATC)_6_	F:AGTCGATCGGGTGTGCTTC	272 - 286	HEX
		R:GAACGAGGGACGGATTTGC		
**Bbf201**	(CT)_8_	F:TCTCTCCACCGCAGGATTG	237 - 254	ROX
		R:ACTGCTGCTCCGAGATACC		
**Bbf219**	(AG)_7_	F:TGAAACTGACCATCGACGC	233 - 239	HEX
		R:GGCAGCCTCCTTCCACAG		
**Bbf250**	(AG)_8_	F:CTCCGACAGTTCGAAGTCC	228 - 239	FAM
		R:CGAAGCTCCGATTGGTACG		
**Bbf271**	(CT)_7_	F:TACTGCGTTACGGCCCTC	172 - 176	FAM
		R:CGATGACTTCAAGCGCCAG		
**Bbf283**	(ACC)_5_	F:AACCGCAATCCCTCACCAG	390 - 397	FAM
		R:CCTTGTTGACTCAGATTCCCG		
**Bbf289**	(AGC)_5_	F:TCAACCACAGAAGTGCCAG	205 - 221	FAM
		R:ATGGAAGCCCGGAATGGAG		
**Bbf377**	(ACTC)_6_	F:AGATTACCATTGCTGCAGACC	205 - 374	TAMRA
		R:AAACAAGAAGCCGGAACCC		
**Bbf384**	(ATGT)_4_	F:ACCGAGTGCACCCTTATAATC	263 - 434	TAMRA
		R:ACAACTTGCACTTCGTGGC		

The PCR amplification reaction system (10 µL) consisted of: 5.0 µL of 2 × Taq PCR Master Mix, 1 µL of genomic DNA (~20 ng), 0.5 µL of forward primer (10 pmol/µL), 0.5 µL of reverse primer (10 pmol/µL), and 3.0 µL of ddH_2_O.The reaction conditions included an initial denaturation at 95°Cfor 5 min, followed by a cycling program of denaturation at 95°Cfor 30 s, annealing at a gradient temperature (62−52°C) for 30 s, and extension at 72°C for 30 s. The cycling program included 10 cycles with a decrease of 1°C per cycle, followed by 25 cycles at a constant annealing temperature. PCR products (3 µL) were analyzed by 1% agarose gel electrophoresis, and their concentrations were estimated by comparison with DNA markers. All samples were then normalized to similar concentration ranges before fluorescence capillary electrophoresis analysis. Prior to fluorescence capillary electrophoresis on a ABI 3730XL sequencer, 10 µL of Hi-Di formamide and 0.5 µL of an internal standard were mixed thoroughly, and 1 µL of the diluted PCR product was added.

### Determination of *l*-Borneol content

According to the previous method [3], precision weighing standard l-Borneol was prepared as reference standard reserve solutions at mass concentrations of 1.031 7 mg/mL. 2 g sample of the material from each of the 51 accessions was extracted with ethyl acetate (25 mL) under ultrasonic irradiation (40 KHz, 400 W) for 30 min. The ethyl acetate extract was then passed through a Millipore filter (0.22 µm) prior to being analyzed by GCs.

The filtered extracts were analyzed on an Agilent 7890A gas chromatograph equipped with a flame ionization detector (FID) and an Agilent G4513A automatic sampler. A HP-5 quartz capillary column coated with a 0.25 µm film was used to analyze the samples. The column temperature was maintained at 80°C for 2 min after injection, and then programmed to increase to 100°C at a rate of 5°C/min. The column temperature was subsequently increased to 200°C at a rate of 20°C/min. The injector and detector temperatures were set at 220 and 240°C, respectively. The system was operated in the split injector mode with a split ratio of 9:1. Nitrogen was used as a carrier gas with a flow rate of 25 mL/min, and the injection volume was set at 0.6 µL.

### Quantification of five flavonoid constituents

Referring to the previous method [4], accurately weigh 5 flavonoid standard substances TMF, Eriodictyol, TDF, Blumeatin and Sakuranetinare to prepare mixed reference solutions with mass concentrations of 1.02, 0.241, 0.203, 0.205, 0.016 mg/mL, respectively. 0.5g sample of the material from each of the 51 accessions was extracted with 90% methanol (10 mL) under ultrasonic irradiation (40 KHz, 400 W) for 45 min. The 90% methanol extract was then passed through a Millipore filter (0.22 µm) prior to being analyzed by HPLC.

The filtered extracts were analyzed on an Agilent 1260 high performance liquid chromatograph with Agilent TC-C18 column (250 mm × 4.6 mm, 5 µm) chromatographic column. The mobile phase was acetonitrile (A) – 0.5% phosphoric acid solution (B), gradient elution (0–15 min, 15% − 22% A; 15–21 min, 22% − 24% A; 21–30 min, 24% − 30% A; 30–45 min, 30% − 33% A; 45–55 min, 33% − 38% A; 55–70 min, 38% − 55% A); Detection wavelength 285 nm; Column temperature 35°C; Volume flow 0.9 mL/min; Injection volume 10 μL.

### Statistical analysis

Excel software was used to analyze the original data, and SPSS 24 was used to analyze the differences. Popgene 32 and PowerMarker 3.25 [20–22] calculated the number of alleles (Na), number of effective alleles (Ne), expected heterozygosity (He), observed heterozygosity (Ho), Shannon index (I), polymorphism information content (PIC), other genetic diversity indicators (PIC is estimated as $PIC=\sum_{i=1}^{k}p_{i}^{2}-\sum_{i=1}^{k-1}\sum_{j=i+1}^{k}2p_{i}^{2}p_{j}^{2}$, $p_{i}$ and $p_{j}$ represent the occurrence frequency of the j band type i respectively, PIC is between 0 and 1, the larger PIC is, the higher the polymorphism of primer is).The Nei’s genetic similarity coefficient was calculated by Ntsys [23]; Cluster analysis was performed using the UPGMA method based on Nei’s genetic distances calculated from SSR binary data (0/1 matrix) with NTSYS-pc software [23]. The cluster diagram was constructed using MEGA [24]. Principal component analysis (PCA) was conducted based on the genetic distance matrix to generate a two-dimensional scatter plot visualizing principal component distributions.

Population structure analysis was performed using Structure 2.3.3 [25]. The number of populations (*K*) was set from 2 to 8, with each *K* value run 5 times. The burn-in period was set to 100,000. The optimal *K* value and membership coefficients (*Q* values) were determined using Structure Harvester (https://taylor0.biology.ucla.edu/structureHarvester/). Germplasms with *Q* ≥ 0.6 were assigned to the corresponding subpopulation. The molecular variance analysis (AMOVA) and principal coordinate analysis (PCoA) were performed using GenALEx 6.502 [26].

The linkage disequilibrium (LD) levels and support probabilities between SSR loci combinations were calculated using TASSEL 2.1 software, and an LD pairwise detection matrix plot was generated [17]. Association analysis between six active component contents and SSR markers was conducted on 51 *B. balsamifera* germplasms using TASSEL 2.1 software. Both the general linear model (GLM) and mixed linear model (MLM) were applied to calculate *P* values and *R*^2^ values. Marker-trait associations were considered statistically significant at *P *< 0.05 and highly significant at *P* < 0.01 [17].

## Results

### Variability analysis of quality traits in *B. balsamifera*

The contents of *l*-Borneol, TMF, Eriodictyol, TDF, Blumeatin and Sakuranetin in 51 samples were determined (Table 3). Results indicated that the coefficients of variation (*CV*) for six bioactive compounds all exceeded 50%, with eriodictyol showing the highest *CV* (85.5%) and TDF the lowest (53.1%). This substantial variation demonstrates significant intraspecific chemical variability in *B. balsamifera* germplasm, supporting its suitability for association analysis targeting these metabolites.

**Table 3:** Statistical analysis of six main quality traits for *B. balsamifera.*

Quality traits	Amplitude of variation	Mean	*SD*	*CV*/%
***l*−Borneol/ (mg·g**^**−1**^)	1.191–14.463	5.833	3.576	61.3
**TMF/ (mg·g**^**−1**^)	1.098–16.086	4.824	3.292	68.2
**Eriodictyol/ (mg·g**^**−1**^)	0.118–3.724	1.116	0.954	85.5
**TDF/ (mg·g**^**−1**^)	0.500–3.540	1.527	0.810	53.1
**Blumeatin/ (mg·g**^**−1**^)	0.363–3.644	1.505	0.846	56.2
**Sakuranetin/ (mg·g**^**−1**^)	0.010–0.225	0.055	0.036	65.0

### Genetic diversity analysis based on EST-SSR markers

**Fig 1:**
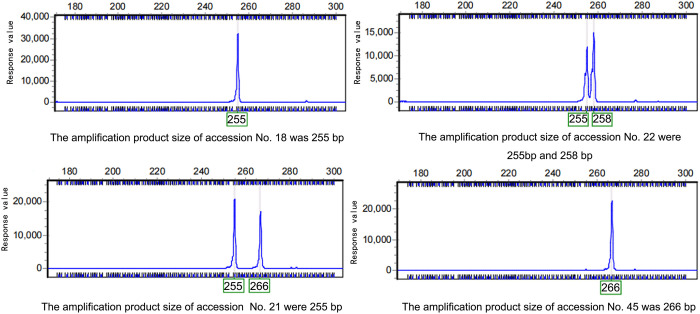
Capillary electropherogram of polymorphic SSR fragments amplified by primer Bbf103 in *B. balsamifera.* Representative capillary electrophoresis profiles of SSR fragments amplified by primer Bbf 103 in selected Blumea balsamifera accessions. Three polymorphic alleles (255 bp, 258 bp, 266 bp) were consistently detected across all samples.

The 51 accessions were amplified using the 22 selected primer pairs. Subsequent capillary electrophoresis with fluorescence detection precisely determined allele sizes across all loci and generated corresponding electropherograms, with representative profiles shown in Fig 1. A total of 102 alleles were detected, with an effective allele number ranging from 1.329 to 4.682 (average of 2.482). The Shannon index ranged from 0.447 to 1.793, while the polymorphism information content (PIC) ranged from 0.222 to 0.762 (average of 0.488). Notably, 41% of the primers exhibited high polymorphism (PIC > 0.5), and 11 pairs of primers showed moderate polymorphism (0.25 < PIC < 0.5). The observed heterozygosity (Ho) averaged 0.494, slightly lower than the expected heterozygosity (He) of 0.548, indicating a moderate level of heterozygosity. These results demonstrate the high polymorphism and genetic diversity of the germplasm, making it suitable for association analysis of quality traits (Table 4).

**Table 4:** Polymorphism of EST-SSR markers.

Primer	Na	Ne	Ho	He	Nei	I	PIC
**Bbf001**	6	1.555	0.333	0.360	0.357	0.784	0.341
**Bbf020**	4	2.257	0.373	0.562	0.557	0.979	0.500
**Bbf041**	3	2.164	0.500	0.543	0.538	0.848	0.437
**Bbf065**	3	1.884	0.569	0.474	0.469	0.700	0.368
**Bbf068**	6	3.814	0.863	0.745	0.738	1.530	0.699
**Bbf070**	7	3.197	0.667	0.694	0.687	1.411	0.644
**Bbf103**	4	1.330	0.196	0.251	0.248	0.536	0.237
**Bbf106**	7	3.752	0.551	0.741	0.733	1.538	0.695
**Bbf137**	4	2.116	0.588	0.533	0.527	0.823	0.416
**Bbf140**	3	1.329	0.245	0.250	0.248	0.447	0.222
**Bbf146**	5	2.135	0.392	0.537	0.532	1.066	0.499
**Bbf156**	3	1.743	0.490	0.430	0.426	0.711	0.363
**Bbf161**	4	3.113	0.765	0.686	0.679	1.241	0.623
**Bbf193**	3	2.112	0.353	0.532	0.527	0.858	0.443
**Bbf201**	8	4.682	0.765	0.794	0.786	1.793	0.762
**Bbf219**	4	2.553	0.438	0.615	0.608	1.049	0.530
**Bbf250**	7	4.439	0.686	0.782	0.775	1.618	0.740
**Bbf271**	3	1.783	0.216	0.444	0.439	0.711	0.365
**Bbf283**	4	1.559	0.392	0.362	0.359	0.689	0.326
**Bbf289**	4	1.968	0.333	0.497	0.492	0.947	0.456
**Bbf377**	6	2.841	0.628	0.654	0.648	1.303	0.605
**Bbf384**	4	2.271	0.529	0.565	0.560	0.933	0.465
**Total**	102	54.595	—	—	—	—	—
**Mean**	4.636	2.482	0.494	0.548	0.542	1.023	0.488
**SD**	1.59	0.969	0.186	0.159	0.157	0.372	0.154

Note: Na, number of alleles; Ne, number of effective alleles; Ho, observed heterozygosity; He, expected heterozygosity; PIC, polymorphic information content.

### Cluster analysis and principal component analysis (PCA) based on EST-SSR markers

**Fig 2:**
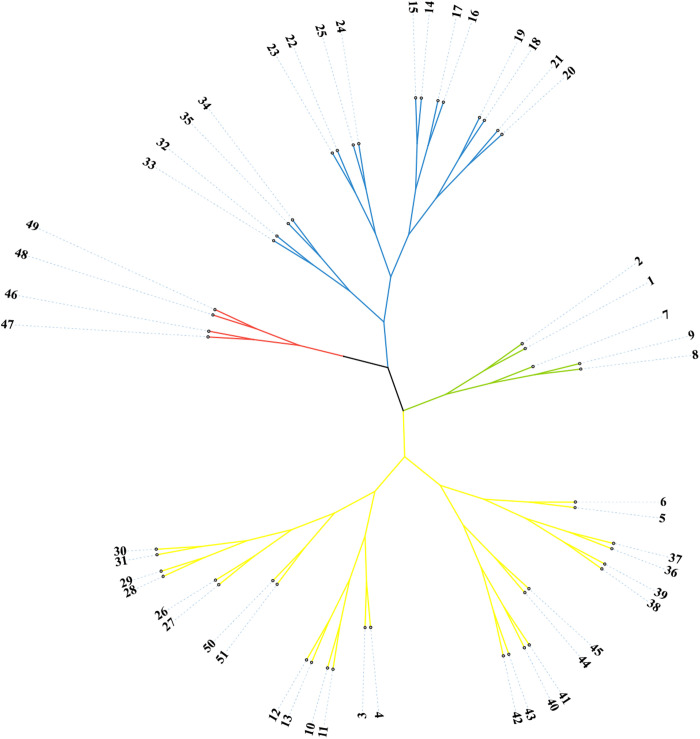
UPGMA clustering of *B. balsamifera.*

Based on Nei’s genetic distance, cluster analysis was performed using the UPGMA method to classify the accessions (Fig 2). At a probability of 0.26, the 51 accessions were divided into four groups. Group I comprised accessions from Guangdong and Danzhou (Hainan), while Group II aggregated materials from Luodian (notably 11 accessions forming a distinct subcluster), Tianlin, Xilin, Zhenfeng, and Danzhou. Group III included accessions from Guangzhou, Nanning, and Wangmo. Crucially, Group IV exclusively contained Hainan accessions from Qiongzhong and Tunchang, demonstrating strict geographic isolation. These findings suggest a correlation between genetic relationships and geographical distribution.

**Fig 3:**
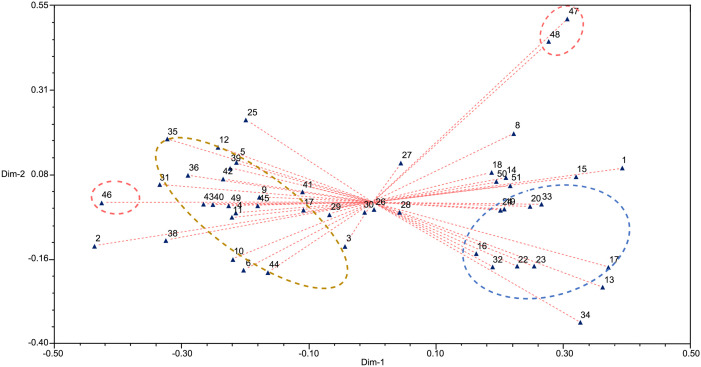
Two-dimensional scatter diagram of principal coordinate analysis of EST-SSR markers.

The Nei’s genetic distance matrix obtained was analyzed by PCA using Ntsys software, resulting in two-dimensional scatter diagram (Fig 3). The distribution of germplasms on the map revealed that geographically distant accessions were relatively far apart, while geographically close accessions tended to cluster together. The distribution pattern observed in the UPGMA clustering analysis was also reflected in the PCA results. Notably, No. 47 and No. 48 from Guangzhou, Guangdong, clustered together and were distant from accessions of other provinces. Similarly, most accessions from Guangxi exhibited proximity, as indicated by the yellow dotted circle in Fig 3. Accessions from Tunchang, Qiongzhong, and Danzhou in Hainan also clustered together, as shown in the blue dotted circle in Fig 3. These findings indicate that accessions with similar geographical origins share a similar genetic background and closer genetic relationships, further validating the accuracy of the UPGMA cluster analysis.

### Analysis of the population structure

**Fig 4:**
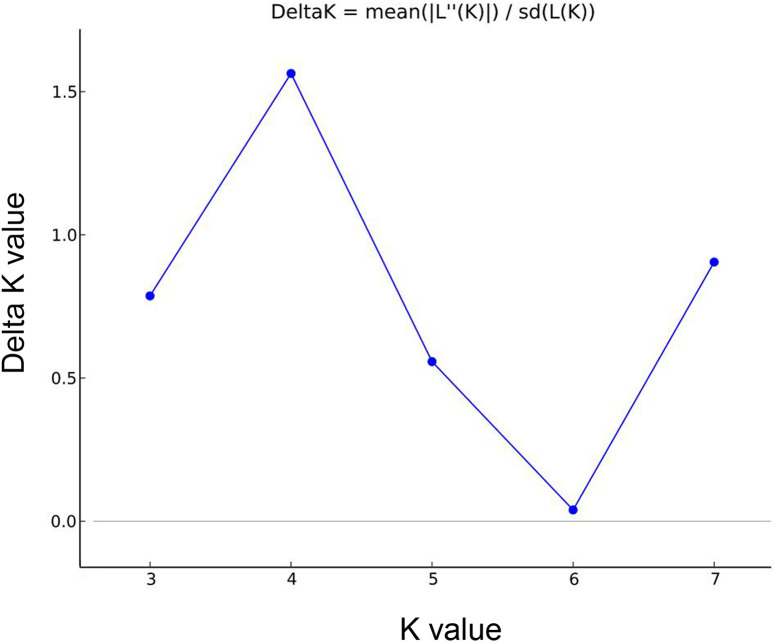
Estimation of the average number of genetic groups using STRUCTURE HARVESTER.

**Fig 5:**
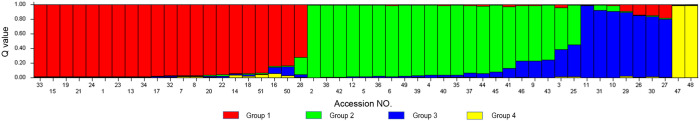
Population structure model of 51 *B. balsamifera* samples based on the genotypic data revealed from EST-SSRs.

The genetic structure of the 51 *B. balsamifera* accessions was analyzed using Structure 2.3.3 software based on genotypic data obtained from 22 primer pairs. The most appropriate subgroup value, *K* = 4, was determined when the allelic variation frequency of the population followed Hardy-Weinberg equilibrium, the sample population was divided into four groups, as depicted in Fig 4. As shown in Fig 5, Q value analysis showed that Q values of all the collected accessions were≥0.6, indicating that the genetic background of these accessions was single, and structural analysis could divide all accessions into four groups. Group 1 (red) consisted of 21 accessions, group 2 (green) had 21 accessions, group 3 (blue) contained 7 accessions, and group 4 (yellow) comprised 2 accessions. Analyzing the germplasm sources, it was observed that accessions from Guangzhou, Guangdong, remained clustered together. Some accessions from Danzhou, Hainan, as well as Tunchang, Qiongzhong, Wanning, and Wuzhishan in Hainan, were concentrated in group 1. No. 1–7 and 12 from Wangmo, Zhenfeng, and Luodian, Guizhou, were predominantly found in group 2. No. 26–31 and No. 10–11 from Luodian, Guizhou, were separately gathered in group 3, possibly due to breeding after group selection. These groupings exhibited significant regional characteristics and were consistent with the classification results obtained from UPGMA and PCA analyses, further confirming the close relationship between genetic relationships and geographical origins of the germplasm.

The diversity information of the four genetic groups was analyzed using GenALEx software (Table 5). The maximum value of number of effective alleles (Ne) was observed in Group 1 (2.254) and the minimum was observed in Group 4 (1.409), with an average value of 1.939 in the overall population. The average Shannon’s information index (I) was 0.688 for the total flax population. The average expected heterozygosity (He) value was 0.4009, which indicated that the population had low genetic consistency and rich genetic diversity. AMOVA showed that 17% genetic variability occurred among the genetic groups, 83% variance was within individuals, the genetic variation among individuals was 0% (Table 6). The fixation index (Fst) for the population was 0.175, which indicated that the large genetic variation differentiation and long genetic distance in the current *B. balsamifera* population. The gene flow among the genetic groups was 1.777 (*Nm* > 1), which explained the significant changes within the genome (Table 6).

**Table 5:** Estimated genetic diversity parameters among the genetic groups as revealed by structure analysis.

Pop	N	Na	Ne	I	Ho	He	uH
**Group 1**	20.773	3.546	2.254	0.871	0.513	0.488	0.500
**Group 2**	20.909	3.818	2.059	0.855	0.465	0.465	0.476
**Group 3**	7.000	2.727	2.033	0.744	0.546	0.446	0.481
**Group 4**	1.955	1.409	1.409	0.284	0.409	0.205	0.273
**Mean**	12.659	2.875	1.939	0.688	0.483	0.401	0.432

**Table 6:** Analysis of molecular variance among the different genetic groups.

Source	df	SS	MS	Est. Var.	Fst	P (rand≥data)	*Nm*	Percent of variance/ %
**Among Pops**	3	90.254	30.085	1.138	—	—	—	17
**Among Indiv**	47	249.619	5.311	0.000	—	—	—	0
**Within Indiv**	51	275.500	5.402	5.402	—	—	—	83
**Total**	101	615.373	—	6.540	0.175	0.001	1.777	100

### Linkage Disequilibrium (LD) of EST-SSR markers

**Fig 6:**
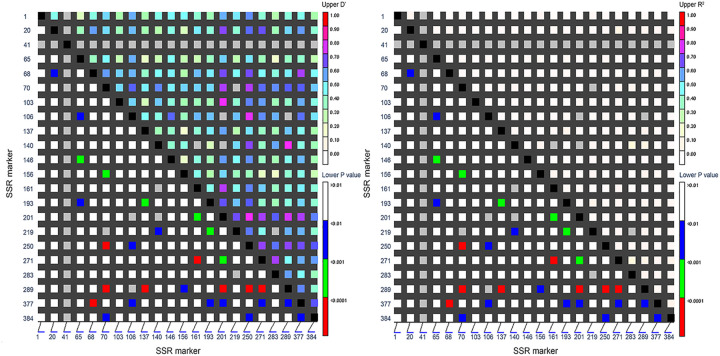
Distribution of LD among 22 EST-SSR loci on 51 *B. balsamifera* accessions. The X- and Y-axes represent sequential SSR locus positions. Pixels above the diagonal display pairwise D’ and R² values, while those below the diagonal indicate LD confidence metrics for locus pairs.

Linkage disequilibrium analysis between different alleles is the basis of association analysis. A total of 231 combinations were generated between 22 EST-SSR loci pairwise in this study. Fig 6 showed the results of using two different parameters *D’* and *R*^*2*^ to describe the LD matrix, where *D’* described the recombination history of the population, *R*^*2*^ reflected the mutation history and recombination history of the population. As seen from the figure, the color of the LD matrix described by the two different parameters was lighter and the LD was not strong, but the LD described by the *D’* value was more obvious than that described by *R*^*2*^. Darker shading in the upper triangle of Fig 6A indicates regions of high LD, signifying strong linkage between adjacent markers. *D’* > 0.5 represents a high degree of linkage disequilibrium, in this study, the value of *D’* mainly ranges from 0.3 to 0.7, accounted for 70.13% of all EST-SSR marker pairs, and the value of *D’* > 0.5 accounts for 51.08%. Elevated LD levels versus wild populations likely arise from historical artificial selection/cultivation bottlenecks fixing large linked genomic blocks, selection-maintained functional genes for adaptive traits within these regions, and inherently low recombination rates-particularly in pericentromeric/heterochromatic zones-delaying LD decay. These results suggest that the recombination and mutation probabilities between genes in the selected germplasm materials were low, consistent with the AMOVA results obtained in the population structure analysis.

### Association analysis between the main quality traits and EST-SSR markers in *B. balsamifera*

Using Q value and Q + K value analyzed by population structure analysis as covariates, the general linear model (GLM) and mixed linear model (MLM) of Talssel 2.1 software were used to associate 22 pairs of SSR markers of *B. balsamifera* with 6 active constituents, and to explore the markers associated with active constituents (Table 7). The results show that in the GLM model, 11 pairs of markers were found to be significantly correlated (*P* < 0.05) or extremely significantly correlated (*P* < 0.01) with the content of 5 active constituents, with a range of variation explained from 19.33% to 57.86%. Among them, the association effect between Blumeatin and marker Bbf106 was the best, with the highest variation explained rate (57.86%). In the MLM model, 5 pairs of markers were significantly correlated (*P* < 0.05) or extremely significantly correlated (*P* < 0.01) with the content of 4 active constituents, with a range of variation explained from 20.82% to 42.86%. Among them, the association effect between Sakuranetin and primer Bbf065 was the best, with a variation explained rate of 42.86%. The MLM model obtained fewer association data than the GLM model, and most of the corresponding explanation rates decreased, indicating that compared with the GLM model, the MLM model can avoid some false positive associations and improve the analysis accuracy by simultaneously considering population structure (Q) and kinship (K) [27].

**Table 7:** The EST-SSR loci associated with six quality traits in *B. balsamifera* and their explained proportion of phenotypic variation.

General linear model (Q)				Mixed linear model (Q + K)			
Trait	Allele	*P* Value	R^2^(%)	Trait	Allele	*P* Value	R^2^(%)
***l*-Borneol**	Bbf068	0.047	34.5*	***l-*Borneol**	Bbf201	0.0233	24.53*
	Bbf070	0.027	41.89*	**TMF**	Bbf137	0.0339	23.42*
	Bbf137	0.007	25.77**		Bbf201	0.0405	20.83*
	Bbf161	0.047	29.56*	**Blumeatin**	Bbf377	1.99 × 10^−5^	28.10**
**TMF**	Bbf001	0.020	33.32*	**Sakuranetin**	Bbf065	0.0067	42.86**
	Bbf137	0.018	22.42*		Bbf106	0.0405	20.82*
	Bbf140	0.021	19.33*	—	—	—	—
	Bbf161	0.035	30.89*	—	—	—	—
**Eriodictyol**	Bbf068	0.045	34.68*	—	—	—	—
**TDF**	Bbf001	0.038	30.51*	—	—	—	—
	Bbf020	0.010	30.5*	—	—	—	—
	Bbf377	0.012	42.77*	—	—	—	—
**Blumeatin**	Bbf065	2.86 × 10^−6^	45.13**	—	—	—	—
	Bbf106	4.68 × 10^−4^	57.86**	—	—	—	—
	Bbf271	0.027	20.82*	—	—	—	—
	Bbf377	1.42 × 10^−4^	57.11**	—	—	—	—

**Note:** *: Significant at *P* < 0. 05; **: significant at *P* < 0. 01.

In both GLM and MLM models, Bbf137 was found to be associated with TMF (*P* < 0.05), Bbf377 was associated with Blumeatin (*P* < 0.01), indicating that Bbf137 and Bbf377 were important EST-SSR loci for controlling Blumeatin and TMF content in *B. balsamifera* respectively, and can be further verified and developed as practical markers. Bbf065 and Bbf106 could be detected in both GLM and MLM models, but their associated components were different. In GLM model, Bbf065 and Bbf106 were significantly correlated with Blumeatin (*P* < 0.01), while in MLM model, they were significantly associated with Sakuranetin (*P* < 0.01, *P* < 0.05).This discrepancy may be attributed to: (1) The GLM only accounts for population structure (Q matrix) and may detect spurious associations, while the MLM incorporates kinship (K matrix) to reduce false positive rates. (2) Blumeatin and Sakuranetin share biosynthetic precursors, and Bbf106 likely marks a gene involved in branch pathway regulation: epistatic interactions with Bbf065 promote blumeatin synthesis (detected by GLM), whereas after MLM background correction, its main effect on sakuranetin becomes apparent. This observation aligns with Kliebenstein [28], which revealed the widespread existence of branch-point gene regulatory mechanisms in flavonoid biosynthesis pathways, providing theoretical support for the markers’ potential involvement in regulating different metabolic branches.

## Discussion

### Genetic diversity analysis of *B. balsamifera* based on EST-SSR markers

In this study, we analyzed the genetic diversity and population structure of the *B. balsamifera* germplasm resources using EST-SSR markers. The results demonstrate that SSR markers exhibit higher PIC values (0.488) and genetic diversity (Ho = 0.494, He = 0.548). In contrast, while Pang, et al. [29] and Zhang, et al. [7] reported high polymorphic fragment ratios for AFLP and SRAP markers (99.48% and 97.78% respectively), AFLP demonstrated lower PIC values (0.1914), whereas SRAP’s PIC (0.4381) approximated SSR levels. Since both AFLP and SRAP are dominant markers incapable of distinguishing homozygous from heterozygous genotypes, their resolution is inferior to that of codominant SSR markers. Consequently, SSR markers are more suitable for studies requiring precise genetic information, such as population genetic structure analysis and association analysis, whereas AFLP and SRAP are better suited for rapid assessment of germplasm resource diversity.

The results also revealed that the tested accessions could be divided into four groups based on the genotype data, with a strong correlation between the genetic relationships and geographical distribution of the accessions. This finding was consistent with the results obtained from UPGMA clustering and PCA analyses, and the two-dimensional and three-dimensional PCA maps were highly coincident, which indicated that the principal component analysis clustering was feasible and accurate and could be used to identify the genetic background and genetic relationship of *B. balsamifera* germplasms [30,31]. Because the present study focused on intra-specific variation, no out-group or external reference population was included; therefore, the results should be interpreted as relative measures of diversity within Chinese *B. Balsamifera* rather than absolute estimates across the genus.

### Population structure analysis of *B. balsamifera* based on EST-SSR markers

Population structure analysis is a prerequisite for association analysis, as overly complex population structures can lead to false positive associations. Thus, simplified population structures facilitate the acquisition of reliable association results [32]. The population genetic structure analysis in this study divided all the samples into 4 groups, and population structure is relatively simple and clear. The single group structure can effectively improve the reliability of association analysis results. Group 1 exhibited the highest number of effective alleles, while Group 4 had the lowest. The average expected heterozygosity (He) value indicated low genetic consistency and rich genetic diversity within the population. AMOVA results showed that a significant portion of the genetic variability occurred among the genetic groups, supporting the presence of genetic differentiation among the geographically distributed *B. balsamifera* germplasms. The fixation index (Fst) value indicated substantial genetic variation differentiation and long genetic distances within the current *B. balsamifera* population. The gene flow among the genetic groups was relatively high (1.777), suggesting significant gene exchange.

These results highlight the importance of considering genetic diversity and population structure in the utilization and conservation of *B. balsamifera* germplasm resources.

### Association analysis between quality traits and EST-SSR markers in *B. balsamifera*

The substantial variation in the content of six bioactive compounds in *B. balsamifera* demonstrates its suitability as an ideal germplasm for association analysis. To avoid false positive associations, the Q and Q + K corresponding to each sample of *B. balsamifera* germplasms was taken as the covariate during the analysis, the quality traits were associated based on GLM and MLM models, effectively reducing and eliminating the impact of population stratification on the association results [33,34]. This study was the first time to associate the quality traits of *B. balsamifera* with EST-SSR marker analysis, in which, Bbf137 and Bbf377 were found to be associated with TMF (*P* < 0.05) and Blumeatin (*P* < 0.01) respectively, in both GLM and MLM models, indicating that Bbf137 and Bbf377 were important EST-SSR loci for controlling Blumeatin and TMF content in *B. balsamifera* respectively, and can be further verified and developed as practical markers, which would provide a basis for molecular marks-assisted breeding of *B. balsamifera* varieties with high Blumeatin and 3,3’,5,7-tetrahydroxy-4’- methoxyflavanone content. In addition, Bbf065 and Bbf106 could be detected in both GLM and MLM models, but their associated components were different. the reason was that the influence of population structure (Q value) and kinship (K value) on association analysis was considered in MLM analysis, so the number of association markers obtained by MLM analysis was less, but the results were more accurate, which may also be related to population size [22,35]. Due to the small sample size in this study and the lack of multiple testing corrections, the results may potentially include false positives. In subsequent work, we will expand the sample size, screen more primers, refine the analytical methods, and validate the marker-trait associations in larger independent populations [36]. Notably, our preliminary analyses identified several high-potential candidates, including Bbf377 (Blumeatin, GLM and MLM *P* < 0.01) and Bbf137 (TMF, GLM and MLM *P* < 0.05). However, their practical breeding utility must be further verified across diverse genetic backgrounds and environmental conditions.

## Conclusions

This study successfully analyzed the genetic diversity and population structure of 51 *B. balsamifera* accessions using 22 EST-SSR markers. The results revealed high genetic differences and rich genetic diversity among the accessions. The high polymorphism and strong discrimination ability of the primers allowed for accurate evaluation of the genetic diversity of all accessions. The UPGMA clustering divided the accessions into four groups, which showed a correlation with the geographical origin of the accession. The PCA analysis indicated that accessions with similar geographical origins shared similar genetic backgrounds and closer genetic relationships. The population structure analysis further confirmed the accuracy of the UPGMA clustering. In the GLM model, 11 pairs of markers were found to be significantly correlated (*P* < 0.05) or extremely significantly correlated (*P* < 0.01) with the content of 5 active constituents, with a range of variation explained from 19.33% to 57.86%. In the MLM model, 5 pairs of markers were significantly correlated (*P* < 0.05) or extremely significantly correlated (*P* < 0.01) with the content of 4 active constituents, with a range of variation explained from 20.82% to 42.86%. In both GLM and MLM models, Bbf137 and Bbf377 were found to be associated with TMF (*P* < 0.05) and Blumeatin (*P* < 0.01) respectively. In summary, this study not only confirmed the feasibility of using EST-SSR markers for genetic diversity analysis in *B. balsamifera*, but also provided a theoretical basis for correlating quality traits with EST-SSR markers in *B. balsamifera* germplasm resources. This study also provides a molecular approach for the molecular identification of *B. balsamifera* germplasm and molecular-assisted breeding of superior germplasm.

## Supporting information

S1 File Raw data. (XLSX)

S2 File Calculation output for SSR genetic diversity analysis. (XLSX)

S3 File Output for genetic diversity, population structure. (XLSX)

S4 File Calculation process and related data of MLM and GLM. (XLSX)
